# The Role of Mobility Digital Ecosystems for Age-Friendly Urban Public Transport: A Narrative Literature Review

**DOI:** 10.3390/ijerph17207465

**Published:** 2020-10-14

**Authors:** Eugène Loos, Maria Sourbati, Frauke Behrendt

**Affiliations:** 1 Utrecht University School of Governance, Utrecht University, Bijlhouwerstraat 6, 3511 ZC Utrecht, The Netherlands; 2 School of Media, University of Brighton, Brighton BN2, UK; M.Sourbati@brighton.ac.uk; 3 Department of Industrial Engineering and Innovation Sciences, Eindhoven University of Technology, PO Box 513, 5600 MB Eindhoven, The Netherlands; f.behrendt@tue.nl

**Keywords:** age-friendly cities, age-friendly urban public transport, smart mobility, age-friendly transport, mobility justice, ICT, older people

## Abstract

Within the context of the intersection of the global megatrends of urbanisation, ageing societies and digitalisation, this paper explores older people’s mobility, with a particular interest in public transport, and a strong consideration of digital/ICT elements. With a focus on (smart) mobility, the paper aims to conceptualise transport, one of the main domains of age-friendly cities as a core element of a smart, age-friendly ecosystem. It also aims to propose a justice-informed perspective for the study of age-friendly smart mobility; to contribute towards a framework for the evaluation of age-friendly smart transport as a core element of the global age-friendly cities programme that comprises mobility practices, digital data, digital networks, material/physical geographies and digital devices and access; and to introduce the term “mobility digital ecosystem” to describe this framework. The paper uses the method of a narrative literature review to weave together a selected range of perspectives from communications, transport, and mobility studies in order to introduce the embeddedness of both communication technology use and mobility practices into their material conditions. Combining insights from communications, mobility and transport and social gerontology with a justice perspective on ICT access and mobility, the paper then develops a framework to study age-friendly smart mobility. What we call a “mobility digital ecosystem” framework comprises five elements—mobility practices, digital data, digital networks, material geographies, digital devices and access to services. The paper contributes a justice-informed perspective that points towards a conceptualisation of age-friendly smart mobility as a core element of the age-friendly cities and communities in the WHO’s global age-friendly cities programme.

## 1. Introduction

“In 2016, there were 512 cities with at least 1 million inhabitants globally. By 2030, a projected 662 cities will have at least 1 million residents.” [1].

“Today, more than half of the world’s population live in cities, with this proportion set to rise to two-thirds by 2050. The global population is also ageing rapidly, with the numbers of people aged over 60 set to pass the 1 billion mark over the next decade. A significant and growing number of the world’s urban residents are older people—more than 500 million.” [2].

These statistics clearly show that two trends, urbanisation and population ageing, are occurring rapidly. Living in an urban area has impact on older people’s everyday life and, for this reason, reports on age-friendly/urban ageing have been published by the World Health Organisation (WHO) (2007, 2018) and the Organisation for Economic Cooperation and Development (OECD) [3]. An important point enhancing older people’s living conditions is to stay mobile because this contributes to their social inclusion. Digitalisation is a third global trend that intersects with urbanisation and ageing societies. Having access to digital elements of and information about transport in urban settings is crucial for older people. The WHO global age-friendly cities programme [4] and the WHO age-friendly environment programme [4] paid attention to age-friendly and inclusive transport attributes, but less so to Information and Communication Technology (ICT).

One can travel in a city by walking, cycling ((e-)bike), motorcycle, mobility scooter, (shared) taxi, (shared/rented/own) car or by using public transport/mass transit (land and water-based). In terms of their broadly similar general mobility patterns, older people travel less than younger people, considering all modes of travel, and often replace driving a private car, after retirement, by walking and the use of public transport, especially buses [5]. Public transport and walking are the most recurring modes of transport among older people, who are less likely to have cars, in cities in Europe [6,7]. Unsuitability or unavailability of service provision in particular areas or for specific destinations [8] can be major barriers to using public transport. The social element of interacting with others on public transport is an important positive element. Musselwhite, Holland and Walker [9] discuss walking, cycling and public transport in relation to health benefits and speculate about the increasing importance of public and community transport for our ageing societies, alongside technological advances that may enable car use later in live (e.g., assisted and autonomous driving). In terms of active transport, there is limited literature that considers walking [10,11] and cycling [12,13,14] and both modes [15]—in relation to old age. Walking is often a key link to public transport, as many public transport users (of all ages) walk to and/or from their access point to public transport [16]—known as “door to vehicle” or the “last mile” portion of the journey.

Many studies on transport and age focus on driving and driving cessation [17,18]. One of the few papers taking a broader perspective has been published by Musselwhite, Holland and Walker [9], going beyond the car in terms of modes and also including virtual mobilities in their approach.

Flexible transport services include demand-responsive transport [19] (DRT) and community transport. The latter is positively regarded, especially by women and the oldest old, but a lack of awareness and information amongst older people is often reported [8]—which is interesting as it has similarities to the “trendy” Mobility-as-a-Service (MaaS) (https://maas-alliance.eu/). Several studies show that walking and cycling were viable options for short trips in the urban context, pointing at the role of the walkability of the built environment [8]. There appear to be less unfulfilled travel needs in urban areas compared to other geographies. Overall, studies in this field reviewed by Luiu et al. [8] “show that at least one-third of older people report unmet travel needs” with “women and the oldest older (75 years old and above)” and those without car access reporting these needs the most [8].

Despite the importance of urban public transport to older people’s mobility in cities—which could be considered as a fundamental right (see Section 3.3)—this area is characterised by a lack of studies [20]. Our paper will critically discuss the intersection of theoretical and empirical studies pertaining to age-friendly urban public transport in developed countries, embedded in what we call a “mobility digital ecosystem”, a notion including also all kinds of digital devices and (access to) services playing a crucial role in this regard (see Section 3.4). Thus, we respond to Marston and Van Hoof’s [21] call for making technologies and ICT central to the WHO’s age-friendly city checklist and to include transport more centrally in their discussions. Their paper reviews a wider range of technology domains such as smart houses, considers technology barriers to use of public transport and the use of delivery robots, alongside a transport-focused case study—all in relation to age.

After having explained the way we have proceeded with our narrative literature review to explore this field in Section 2, we present its results in Section 3.1, Section 3.2, Section 3.3 and Section 3.4. The insights will be used in Section 4 to propose a framework for a research agenda that combines insights into the ways older adults make sense of their mobility digital ecosystem, focusing on older people’s ICT (in)capability in relation to the role of the built urban environment (including technologies and systems of transport/communications) to enhance inclusive mobilities in later life.

In our paper, we will adopt a mobility approach to age, which means that, instead of focusing on functional/utilitarian (economic/engineering) and trip aspects, we will use a broader understanding of mobility that includes social, emotional, embodied and political elements [9,22,23].

## 2. Method: Narrative Literature Review

We conducted a narrative literature review “in which the findings (…) of relevant studies are outlined and discussed with a view to presenting an argument about the conclusions that can be drawn from the current state of knowledge in a field” [24]. Systematic literature reviews, often used in the field of quantitative evidence-based empirical studies, are not well suited to social science topics that explore new topics, and particularly not for multi-disciplinary areas such as the focus of this paper (see e.g., [25]).

Insights from communications [26,27,28] and transport technology use [29,30] and transport mobility practices [31,32] will be used in Section 3.1 as a starting point to explore the materialities and digital elements of communications, mobility and transport. Reports from the World Health Organization and publications such as those by Alley, Liebig, Pynoos and Banerjee [33], Buffel, Handler and Phillipson [34] and Steels [35] will be used to get an insight into transport and mobility in age-friendly cities (Section 3.2). In Section 3.3, we will discuss the importance of mobility rights for older people by building on Sheller’s concept of *“mobility justice*” [36] comprising differential access to spaces, services and social goods as “not just the result” of racial, gendered, classed, sexed, and, in our study, aged formations, but also “as *productive* of those hierarchical systems of differentiation, through various kinds of enablement and disablement” [36]. The studies by Behrendt, Murray, Hancox, Sourbati and Huber [37] and Sourbati and Behrendt [38] will be used to explore how we can move forward to age-friendly smart mobility in Section 3.4. Then, in Section 4, we will use the insights from Section 3.1, Section 3.2, Section 3.3 and Section 3.4 as a conceptual framework to understand the dynamics of the use of digital devices and (access to) services as practices in an urban “mobility digital ecosystem”.

## 3. Literature

### 3.1. Communications, Mobility and Transport as Material and Digital Entanglements

This section weaves together a selected range of perspectives from communications studies, transport studies and mobility studies to introduce the embeddedness of both communication technology use [26,27] and transport mobility practices [31,32] into their material conditions.

Historically, electronic communication technologies and motorised transport have been commonly contextualised in terms of one another [29]. During the interwar period of the 20th century, both new media technologies (radio and moving image) and transportation (the automobile, air travel) were “[c]onsistently paired” in public discourse as the prime agents behind the proliferation of modes of connection and contact between people and places [30,31]. Originating in the early years of communications studies, this perspective on communication as “organized movement and action” [30] has been revisited in the study of more recent developments surrounding mobility and contact as media technology and transportation “remain inextricably linked in ways that are both conceptual and material” [29]. Over the last 10–15 years, transportation, typically understood as a form of organised movement, has embedded digital ICT [39,40]. Research into the ways digital ICTs, in particular the smartphone, have profoundly changed the ways in which transport systems are perceived and used, and mobilities performed, has underlined the contribution of smart apps to the encouragement of sustainable travel [41].

Both communication and transport are entanglements of material and virtual elements, comprising fixed, in-place infrastructures, including roads, railways, pavements, transit stations, bus stops, traffic lights, and Wi-Fi infrastructure; motorised and non-motorised vehicles [5,7,42] communications devices; connectivity, such as mobile internet, street sensors or cameras; systems of licensing and regulation (e.g., governing the provision of telecommunications/network connectivity and the collection, ownership and sharing of digital data [43]), and embodied practices of movement [44]. Moving around the city integrates mediated communication with material forms of movement and action [27,30,45]. Communication can be seen to play an important role in the constitution of socio-spatial territories [28] by combining and integrating the movement of people and information. Thus, mobile media use has profound social consequences as “not only means of communication but increasingly also of generating data” [46] Contextualised in terms of its material surroundings, mobile communications can be likewise seen to “meld with pedestrian urban ecologies” [45]. Within this entanglement, the interaction of walkers, passengers, drivers, riders, etc., combines material infrastructures and mediated or symbolic forms, such as information, data and meanings. Infrastructures, understood as systems that facilitate action in communication [47] and transport [48] are shaped by pre-existing cultural logics that serve to inform, but also “amplify’, and “inflect” their adoption and contemporary uses, often producing stratified, mediated connections [28].

Social imaginaries of age [49] are an example of how cultural logics have historically informed the design of infrastructures and technologies in transport systems [31], pedestrian movement and journey planning applications [50] and, more generally, ICT design [51,52], smart mobile telephony [53,54,55] and Artificial Intelligence (AI) [56].

Within a broader context of wider social changes such as the growing and ageing urban populations that overlay and interact with mobility [5], the needs of older city residents are beginning to receive some acknowledgement across Europe as an area of smart city policy outcomes [57] but these are yet to be addressed by stakeholders. In this socio-economic context, the digital ICT capabilities of older groups and the growing diversity of ageing urban populations constitute an important conversation to be held on the future of urban mobility—and of the city [58]. In Section 3.4, we will focus on the specific role of digital devices for (access to) urban public transport services.

### 3.2. Transport and Mobility in Age-Friendly Cities

The creation of age-friendly environments worldwide has been promoted by the World Health Organization (WHO) [59,60] who define an “age-friendly” city as a city that “optimiz[es] opportunities for health, participation, and security in order to enhance quality of life as people age” [59]. Transportation, including accessible and affordable public transport, is a key theme in the WHO age-friendly city checklist [59]. Alley, Liebig, Pynoos, Banerjee and Choi [33] underline the involvement of older people in age-friendly environments and describe age-friendly cities and communities as “places that actively involve, value, and support older adults, both active and frail, with infrastructure and services that effectively accommodate their changing needs” while Buffel, Handler and Phillipson [34] underline the significance of finding ways older people themselves can be involved in the co-production of age-friendly policies and practices. For more information about the characteristics of age-friendly cities, see also [21,61].

Age-friendly cities and communities (AFCC) [60] have become an important area of work in the field of public policy and ageing, reflecting the importance of the physical and social environment in maintaining the quality of life of older people, given the complexity of demographic change [61] in the context of global “megatrends” [62,63] that also include rapid urbanisation, digital ICT and climate change. The age-friendly agenda has resulted in an increased discussion of problems facing older people living in urban environments as well as of strategies and initiatives which ensure policies, services and products meet the needs of older persons [21,35]. As population aging becomes a widely noted “issue-fied”matter [64] that is granted urgency, significant political motivations are emerging to pursue “age-friendly” community initiatives [65]. This growth in interest in “age-friendly” issues by public policy and stakeholders has stimulated new approaches to the built environment, housing and neighbourhood design that highlight the importance of physical contexts [66]. Information and communication technology (ICT) has been integral to these approaches [5,7,42].

With AFCC debates and initiatives now taking off, social policy and critical gerontology scholars are highlighting the need to integrate research with policy [34] for age-friendly strategies to promote the health and wellbeing of older adults [67]. Applying a more inter-generational perspective, sustainable all age-friendly models [68,69] can create urban spaces and places inclusive of social and emotional aspects of intergenerational belonging and community [58]. From the perspective of mobility (and transport), we understand age-friendly models to embrace social and environmental citizenship in addition to social policy goals, including safe and sustainable travel and connection to other people.

**Figure 1:**
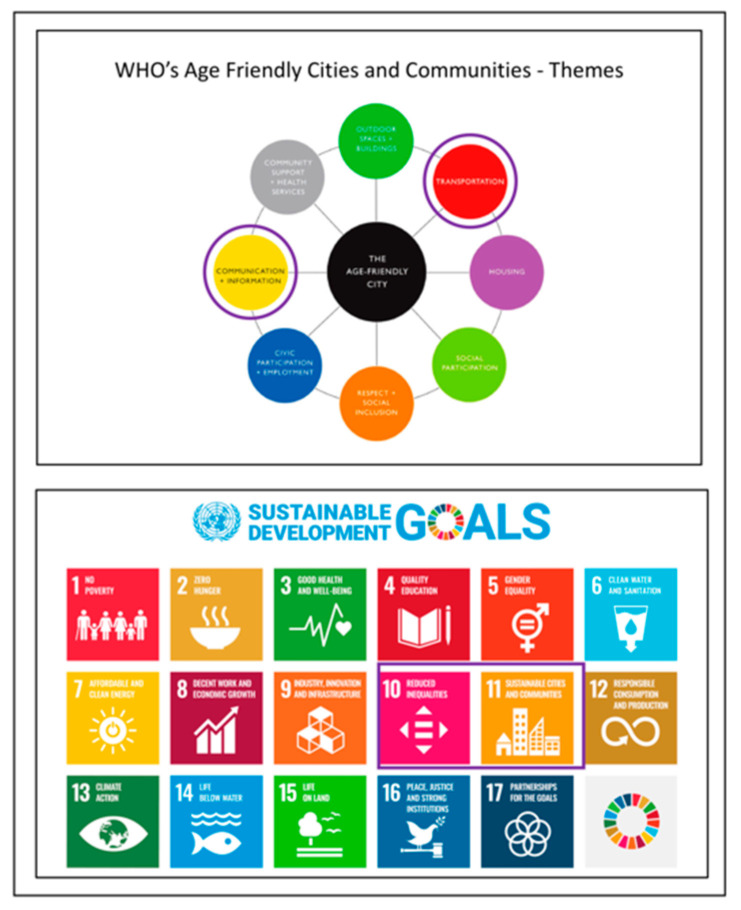
The two key themes of the WHO’s Age-Friendly Cities and Communities and of the UN’s Sustainable Development Goals (highlighted in purple) most relevant to this paper. Author’s illustration, based on illustrations by [72] and by [73].

Transport and mobility, both in their traditional forms as well as in “smart” mode, should, therefore, be seen as a core component of age-friendly cities and communities. Transportation has been highlighted by the WHO [59] as a key factor that influences active and healthy aging, and so is “Information and Communication” (see also Figure 1). Smart Transport is arguably situated at the intersection of these two topics—as it refers to the use of digital technologies to improve transport by improving access to information about any aspect of the journey, including destination and pickup points, booking and payment systems, timetable, etc. Smart Transport can enhance age-friendly outcomes, such as the ability to move about the city, which in turn “determines social and civic participation and access to community and health services” [59]. According to Gassmann, Böhm and Palmié [70], smart mobility pursues sustainable, innovative, and secure transportation systems; access to diverse transportation modes; good availability in the entire city; inclusion of nonmotorized transportation; integration of ICT in transportation systems (see also Section 3.4, where the concept of smart mobility will be discussed more in detail). These objectives can promote the “age-friendly” agenda [59,60] based on inclusive ICT: we propose the concept of all age-friendly transport (similar to Murray’s [22] argument) where the design and deployment of digital ICT and data are steered towards the creation of tools to support inclusivity, e.g., by widening the participation of excluded older groups who are typically the most excluded in the digital economy (though not the only ones), and sustainability. A focus on transport and mobility can highlight broader areas of impact of the converging trends in digital ICT, datafication, mediatisation, climate and demographic change [38]. Accordingly, we conceptualise age-friendly infrastructures as smart, green, public, inclusive, sustainable, and safe. This approach links the WHO’s Age-Friendly Cities and Communities to the UN sustainability goals, specifically the goals “reduced inequalities” and “sustainable cities and communities” [71]; see also Figure 1.

### 3.3. Access, Equity and Mobility Justice in the City

If we wish to ensure that older people can continue to participate in our society, then access to services and (digital) information about these services are of prime importance [74]. Van den Hoven [75], referring to Rawls [76,77], goes so far as to refer to accessible information as a “primary good”. As all citizens have an equal right to access to information, Bovens [78] and Bovens and Loos [79] even advocate granting citizens information rights, next to the classic (freedom) rights. It is important to refer here to an initiative to guarantee the rights of older people, The Madrid Plan of Action [80], offering “a bold new agenda for handling the issue of ageing in the 21st-century. It focuses on three priority areas: older persons and development; advancing health and well-being into old age; and ensuring enabling and supportive environments.” See also the related UN Report of the Second World Assembly on Ageing [81] and the Follow-Up to the Second World Assembly on Ageing [82].

In the context of access to mobility and transport services (and information about them), cultural age-related bias remains a major impediment to age-friendly city initiatives. Older people have been systematically excluded from participation in decision making [34,83] and continue to remain invisible in research and datasets [38,56]. In order to widen the participation of excluded groups such as older (and younger) pedestrians and public transport users in transport and social policy, it is therefore important to focus on age as a social category in its own right while simultaneously recognizing social inequalities within the older adult population [84].

Enhancing the participation of excluded groups and their access to services points towards a conceptualisation of smart mobility as a core element of the age-friendly city that aims to frame smart transport mobility [34,85] as an area for debate and policy intervention based on a better understanding of the mobility activity, transport use and travel patterns [31,86], and mobile media use patterns of a diversity of (older) groups and how these are developing during their life course. While mobility and/or digital connectedness are key to reaching many opportunities, it is important not to conflate mobility (or digital connectedness for that matter) with accessibility.

Attuned to human rights and freedoms perspectives [31], access to ICT connectivity, destinations, and modes of transport foregrounds questions of resource (re)distribution, equity and justice, as captured in evolving discussions of transport and mobility justice [39,87,88]. Mobility is “a prerequisite for citizens to have independence and participate in activities, access services, and form social relations” [31]. Accordingly, differential access to spaces, places, services and social goods through transport systems are “not just the result” of inequality but are “also *productive* of [...] hierarchical systems of differentiation, through various kinds of enablement and disablement” [39].

Fainstein’s [89,90] concept of a “just city” and her work to identify approaches to realizing it within the urban context can provide a bridge between justice-oriented perspectives on mobility, transport and the city.

To assess implementation of policy goals, justice-oriented research into mobility [91], transport [92] and ICT [93] has been drawing on the capabilities approach (CA) as developed by Sen [94] and Nussbaum [95]. The concept of capabilities for wellbeing has been applied here to study policy responses to the needs, experiences and practices of diverse groups. Capabilities define the sets of freedoms and opportunities available for individuals to choose and to act to fulfil their basic potential. At a minimum, these comprise rights to life, health, bodily integrity, access to education, and the ability to participate politically and materially in shaping one’s environment [94,95]. Conceptualising capabilities as an interface of situated individuals and contextual factors [96] helps to identify areas for policy intervention to address the social basis of capabilities [95,97] and strategies to influence the opportunities and overcome barriers. Since the 1990s, the capabilities approach has provided a well-reasoned case, based on a justice perspective for the promotion of the UN’s Millennium Goals [98] and Sustainable Development Goals (SDGs) [99], linked to the WHO’s definition of Healthy Ageing [100,101]. In this paper, we take a capability approach to the study of mobility in old age based on a multidimensional understanding of accessibility that acknowledges the diversity of people’s needs and constraints [31,91,92]. A “capability approach” offers a strong conceptual framework to assess the implementation of policy goals, including those surrounding age-friendly cities and communities (AFCC) and, more generally, the progress of the Sustainable Development Goals (see also Figure 1). Like the SDGs, the capability approach takes a multi-dimensional perspective, is applicable to all societies, shows concern for “all people, everywhere”, and has interconnectedness across dimensions at its core [102].

### 3.4. Towards Age-Friendly Smart Mobility

The studies by Behrendt, Hancox, Huber, Murray and Sourbati [37] and Sourbati and Behrendt [84] into the role of inclusive public transport address wider societal challenges such as loneliness and isolation, civic participation, connectivity and health and wellbeing in relation to mobility and the physical–environmental context. Here, they are brought into conversation from the perspective of age-friendly, inclusive transport, especially the topic areas of “transportation” and “information and communication” in the WHO Global age-friendly cities programme [5] (https://www.who.int/ageing/projects/age_friendly_cities/en/) to highlight the importance of smart mobility and transport for the social inclusion of ageing urban populations.

Jeekel [102] discusses smart mobility in the context of those who are involuntarily “transport disadvantaged” by having greater efforts in getting to locations that are relevant for their regular activities, either through distance or through “forms of disability”, which, for him, include age [103]. Out of the four smart mobility elements he identifies—vehicle technology, intelligent transport systems, data, and mobility as a service—he highlights that the potential of data for public transport is currently not very well realised, while automated driving is likely to serve those older people who are not disadvantaged [103]. Jeekel [102] sees the best potential in mobility as a service. However, he also cautions how this will be financed for the transport disadvantaged, how prices will be set, how subsidies will work, and also how MaaS would be replacing or complementary to public transport [102]. We agree with Jeekel [102] that it is important to be aware that “digital literacy” is required to engage with mobile devices for booking MaaS. Being able to buy and handle user-friendly digital devices for (access to) services for urban public transport is crucial (see also Section 4).

Battarra et al. [103] consider a number of projects to identify seven categories of smart mobility measures for older citizens (improvement of public transport, improvement of public transport comfort, improvement of road network, ITS for private transport, promotion of soft mobility, promotion of shared mobility, implementation of info-mobility services) and analyse Italian cities (across several parameters each for safety, accessibility, ICT). However, while some ICT items measured might be useful for older inhabitants, e.g., electronic bus stop signs, others might instead act as barrier, e.g., electronic travel tickets on mobile devices, and, in that sense, can be considered as enabling constraints [104].

The social element of mobility is also a key consideration in the context of ageing societies. Jeekel [102] calls for creating “a transport system that maximises the possibility to meet, via mobility, all sorts of people, and is basically about the power of joint experiences, dialogue, and creating community via transport and transport services” [102]. This is an issue relevant for both ICT and mobility, but is often forgotten at their intersection.

Just as cycling is often excluded from “smart mobility” industry and policy agendas in favour of a car-centric approach [105] it would be pertinent to analyse if the same is true for older people (in favour of those of “working” age). National policies on transport and mobility need to take an inclusive approach [103], including considerations around (older) age. The same is true for ICT/digital policies. The quickly growing intersection between both—where mobility and smart technologies merge—needs to be high on policy agendas to make inclusivity and age diversity key requirements and features of current and future smart mobility developments, to channel the rapid industry development (in both the digital and transport sector) in these areas towards inclusive, sustainable and just cities and societies.

## 4. Mobility Digital Ecosystems in Later Life: Framework for a Research and Policy Agenda

The preceding discussion shows the importance of integrating the examination of continuities in media technologies and transport systems, the socio-cultural logics informing their design, and changes made possible by digital connectivity and data in order to understand the affordances of (smart) transport systems in promoting or impeding mobility for older people as well as younger age groups. Drawing on capability analysis, transport and mobility were discussed as person–environment, interactive and resource-dependent practices, highlighting the role of public policy in shaping access to those systems. The policy, the design, the spatial, the personal and the social elements can be understood as interconnected in the provision of opportunities to access transport and mobility capacity.

**Figure 2:**
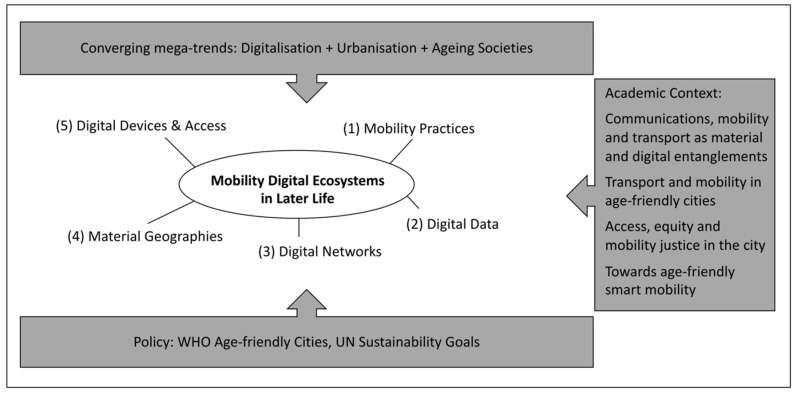
The framework “Mobility Digital Ecosystems in Later Life” and its five elements, within the larger societal (top), policy (bottom) and academic (right) contexts.

In this final section, we will use these insights in a framework for developing research and policy that recognises the importance of smart transport as an integral element of smart, age-friendly cities of the future (see also Figure 2). We coin the term “mobility digital ecosystem” for this framework; see also Marston and Van Hoof’s [21] concept of a “digital ecosystem”, proposed for a critical discussion of the WHO’s notion of an “age-friendly city”. We put the word “mobility” in front of their “digital ecosystem” to highlight how the cultural, social and political context of public urban transport used by older people increasingly intertwines digital and mobility elements. The mobility digital ecosystem is composed of five elements—mobility practices, digital data, digital networks, material geographies, digital devices and (access to) services—as follows:

(1) Mobility has been broadly defined in relation to the embodied practices of moving around (Merriman and Pearce [44] and the wellbeing of individuals, as the ability to choose where and when to travel and which activities to participate in outside the home in everyday life [91]. Mobility practices integrate personal and environmental components [31] and combine material and digital objects [106]. Digital, mobile ICTs such as the mobile phone not only support social connectivity when their users are in physical motion [107] but also support movement in place, through the production and use of digital data [89]. ICT and data infrastructures are increasingly integrated into an expanding range of mobility including walking, velomobility/cycling [106] and other non-motorised means of transport, as well as electrically assisted modes of transport including bicycles, cargo bicycles and freight vehicles, push scooters, skateboards, trikes and personal mobility devices alongside motorised vehicles such as private cars, car sharing and public transport. All these modes describe complexes of social practice (including working, shopping, leisure, visiting friends and family) which are embedded into their material conditions [31] and connected to infrastructural arrangements across space and time [108] (e.g., routes, destinations, shelters, data infrastructures) and included or excluded [109] in ways that cannot be controlled by individuals alone. Thus, mobility can usefully be analysed as a “person–environment relationship” (see, e.g., [110]) of transport mobility, comprising the physical/geography of places and the built environment, the social/cultural and the institutional/regulatory systems [111,112]. These practices of mobility and experiences of age shape and influence each other. Older people’s mobility patterns are changing but not in a homogenous manner. With mobility widely recognised as an important factor in older people’s wellbeing, Levin emphasises the need to consider variations in experiences of age and mobility practice among older groups, including in health and fitness and places of residence, in the application of recurrent concepts of wellbeing and independence. Multi-modal mobility can therefore play a key role in encouraging the use of public transport along more active modes in the transition from automobility, including after the cessation of driving licenses.

(2) Digital data: As the range of digital ICT applications is expected to increase, our cities and transport systems become more instrumental in providing data for digital AI application development [113], combined with data generated through mobile ICT use [38]. Accordingly, both the role of this data and our expectations and use of AI are bound to increase. Age-friendly smart public urban transport requires algorithms that are capable of setting up bias-free training datasets and statistical models capable of incorporating the digital media practices of broader population segments [56].

(3) Smart mobility relies on digital networks, in addition to mobile physical objects and people, while smart mobility involves data collection and analysis on a scale [114]. Smart mobility systems combine physical, digital and data infrastructures. These comprise intelligent transport systems, where networked ICT capability is applied in existing mobility systems, including sensors in public roads and parks, Internet of Things (IoT) solutions built into public and private transportation modes such as buses and cars, and citizens’ use of networked ICT (e.g., traffic management), data, and new mobility services [115]. According to Gassmann, Böhm and Palmié [74], smart mobility pursues the core objectives of sustainable, innovative, and secure transportation systems, access to diverse transportation modes, good availability in the entire city, the inclusion of nonmotorized transportation, and the integration of ICT in transportation systems. Expanding the conceptualisations of mobility as a person–environment relationship [115,116] to encompass digital data generation, registration and use, we can develop a socially oriented analysis of smart mobility in urban settings for later life.

(4) Together with material geographies and built environments (such as roads, pavements, parks, city centres, the systems of roads and pavements, traffic lights, Wi-Fi) [7,27], the digital infrastructures of a city have consequences for transport and mobility. Their construction and regulation shapes both the kinds of effects that existing spatial arrangements may have (on transport systems and organised movement) and the new spaces created by smart ICT (see [117]). These also highlight areas of opportunity. Older people are currently underserved by transport systems with physical and digital infrastructures, including the surface of pavements, the provision of benches, seating areas in terminals and bus stops [5], pedestrian crossing lights [117], digital interfaces such as journey planners and maps designed for more able-bodied younger users, and can be seen as discriminatory [5]. Smart transport solutions can make public transport, and community/flexible transport services more accessible by both addressing barriers to access relating to difficulties in getting information about the provision of services, as well as by enabling the signalling of demand for travel services and making viable flexible transport solutions [40] as well as issues relating to security/safety on board [8] In an urban context, promoting opportunities to access mobility entails both physical and social infrastructures of media and transport access.

(5) Digital devices and (access to) services are crucial to enable (e.g., by providing access to transport information) older people’s urban mobility. However, we should beware of the fact that, at the same time, digital devices also risk constraining older people—for example, when people are incapable or not willing to use digital devices, e.g., because they are perceived as too complex or expensive [118,119]. Thus, digital devices can be seen as enabling constraints [104]. It is also important to mention the so called “I-methodology’, which is “the reliance on personal experience, whereby the designers consider themselves as representatives of the user” [120] and, without being aware of it, produce user representations that resemble themselves (designers tend to be younger adults). To enable older people to be mobile in an urban setting as much as possible, it is therefore important to give them a voice by involving them in the co-design of user-friendly digital devices (see [121]) and the co-production of age-friendly digital policies and practices (see [33]).

## 5. Conclusions

Within the context of the intersection of the global megatrends of urbanisation, ageing societies and digitalisation, this paper explores older people’s mobility, with a particular interest in public transport, and a strong consideration of digital/ICT elements. It presents a transdisciplinary and a mobilities approach to age that is a “broader navigation of the experiences of ageing and the ways in which people of different ages experience urban spaces” [22]. The approach integrates the examination of continuities in media technologies and transport systems, the socio-cultural logic informing their design, and changes made possible by digital connectivity and data, in that order. This involves understanding the affordances of (smart) transport systems in promoting or impeding mobility for older people as well as younger age groups.

The paper combined insights from the communications, mobility and transport literature (including [26,27,31,32]) and social–gerontology research on transport, mobility, and age-friendly cities (including Alley, Liebig, Pynoos and Banerjee [33], Buffel, Handler and Phillipson [61], Buffel and Phillipson [34] and Steels [35]). Furthermore, it drew on justice perspectives related to media/technology access, equity and mobility (such as Sheller [88]) and on age-friendly smart mobility [38,103]. The paper also drew on the policy literature and initiatives such as the WHO Global age-friendly cities and communities programme and the UN’s sustainable development goals. Building on these bodies of academic and grey literature, the paper developed the concept of the “mobility digital ecosystem”, which engages with the services and digital information around public urban transport used by older people. This concept forms the backbone of the proposed framework for a research and policy agenda that combines insights into the ways older adults make sense of their mobility digital ecosystem, focusing on older people’s ICT (in)capability in relation to the role of the built urban environment (technologies and systems of transport/communications) to enhance inclusive mobilities in later life. The “mobility digital ecosystem” is comprised of five elements—mobility practices, digital data, digital networks, material geographies, digital devices and (access to) services (see also Figure 2).

This interdisciplinary review has highlighted that smart urban public transport for older people is currently under-researched, despite its growing importance in the context of the urbanisation, digitalisation and ageing of societies around the globe. Future research in this area could include empirical work that draws on the literature and concepts identified in this narrative literature review, such as analysing policy documents at transnational, national regional or local levels, and interviews with older people, as well as relevant policy and industry stakeholders. Furthermore, studies on urban mobilities, public transport, old age, and digital society should ideally take into consideration each of these elements, rather than only focusing on one of them. This is also relevant to research on smart cities. Future research should also consider structural differences between countries and areas to understand in more detail how urban mobility and digital literacy differ for older people, for example, between Northern and Southern Europe, but also on a global scale. The research options sketched out here would also contribute to validating (elements) of the framework we developed in this paper.

The review has also shown how important it is that international, national, regional and local policies on both transport/mobility and ICT take an age-inclusive approach. This could translate into a number of practical policy steps. Funding opportunities as policy tools in the areas of mobility and ICT could stipulate the inclusion of older age groups, for example, as participants in pilots or in design/user experience (UX) approaches, such as personas or user journey maps. This could also be extended to commissioned policy reports, tendering and commissioning. It is important for policy agendas to make inclusivity and age diversity a key requirement and feature of current and future smart mobility developments, in order to channel the rapid industry development (in both the digital and transport sector) in these areas towards inclusive, sustainable and just cities and societies. Overall, this paper contributed a justice-informed perspective that points towards the conceptualisation of smart mobility as a core element of an age-friendly city.
